# Forming 4-Methylcatechol as the Dominant Bioavailable Metabolite of Intraruminal Rutin Inhibits *p*-Cresol Production in Dairy Cows

**DOI:** 10.3390/metabo12010016

**Published:** 2021-12-24

**Authors:** Yue Guo, Wanda J. Weber, Dan Yao, Luciano Caixeta, Noah P. Zimmerman, Jesse Thompson, Elliot Block, Thomas G. Rehberger, Brian A. Crooker, Chi Chen

**Affiliations:** 1 Department of Food Science and Nutrition, University of Minnesota, 1334 Eckles Ave., St. Paul, MN 55108, USA; guoxx390@umn.edu (Y.G.); dyao@umn.com (D.Y.); 2 Department of Animal Science, University of Minnesota, 1364 Eckles Ave., St. Paul, MN 55108, USA; weber023@umn.edu (W.J.W.); crook001@umn.edu (B.A.C.); 3 Department of Veterinary Population Medicine, University of Minnesota, 1365 Gortner Ave., St. Paul, MN 55108, USA; lcaixeta@umn.edu; 4 Church & Dwight Animal and Food Production, Waukesha, WI 53186, USA; Noah.Zimmerman@churchdwight.com (N.P.Z.); Jesse.Thompson@churchdwight.com (J.T.); Elliot.Block@churchdwight.com (E.B.); Tom.Rehberger@churchdwight.com (T.G.R.)

**Keywords:** rutin, quercetin, 4-methylcatechol, *p*-cresol, microbial metabolism, dairy cow

## Abstract

Rutin, a natural flavonol glycoside, elicits its diverse health-promoting effects from the bioactivities of quercetin, its aglycone. While widely distributed in the vegetables and fruits of human diet, rutin is either absent or inadequate in common animal feed ingredients. Rutin has been supplemented to dairy cows for performance enhancement, but its metabolic fate in vivo has not been determined. In this study, plasma, urine, and rumen fluid samples were collected before and after the intraruminal dosing of 100 mg/kg rutin to 4 Holsteins, and then characterized by both targeted and untargeted liquid chromatography-mass spectrometry (LC-MS)-based metabolomic analysis. In plasma and urine, 4-methylcatechol sulfate was identified as the most abundant metabolite of rutin, instead of quercetin and its flavonol metabolites, and its concentration was inversely correlated with the concentration of *p*-cresol sulfate. In rumen fluid, the formation of 3,4-dihydroxyphenylacetic acid (DHPAA) and 4-methylcatechol after rapid degradation of rutin and quercetin concurred with the decrease of *p*-cresol and the increase of its precursor, 4-hydroxyphenylacetic acid. Overall, the formation of 4-methylcatechol, a bioactive microbial metabolite, as the dominant bioavailable metabolite of rutin and quercetin, could contribute to their beneficial bioactivities in dairy cows, while the decrease of *p*-cresol, a microbial metabolite with negative biological and sensory properties, from the competitive inhibition between microbial metabolism of rutin and tyrosine, has the potential to reduce environmental impact of dairy operations and improve the health of dairy cattle.

## 1. Introduction

Flavonoids, as a large group of ubiquitous polyphenolic compounds in plants [1], are well known for their health-promoting and disease-preventing effects in humans and production animals, mainly through their anti-inflammatory and anti-oxidative properties [2]. Quercetin is one of the most bioactive flavonoids. It is widely distributed in the fruits and vegetables of a human diet as the aglycone of rutin (quercetin-3-O-rutinoside), but deficient in common plant-derived feed ingredients of monogastrics and ruminants, such as corn, soybean, and alfalfa [3]. Therefore, the use of quercetin in animal feed, commonly through rutin supplement, has been extensively explored for alleviating the morbidities under pathophysiological challenges. In dairy cows, positive effects of quercetin and rutin on growth, health, reproduction, metabolism, and milk production have been observed [4,5,6,7]. In young calves, feeding quercetin decreased splanchnic glucose oxidation, increased glucose absorption, and increased antioxidative capacity [5,8]. In lactating cows, intraduodenal supplementation of quercetin enhanced insulin release and sensitivity [9], and mitigated the hepatic disorders from lipid accumulation and metabolic stress [6]. Furthermore, the modulation of rumen microbiota was observed after rutin supplementation in dairy cows, as shown by the decrease of methane production [7] and the increases of short-chain fatty acids (SCFAs) and crude protein from fermentation [4]. All these beneficial effects on health and metabolism can be further translated into the enhancement of energy utilization and production in dairy cows.

Many proposed mechanisms on the health- and growth-promoting effects of quercetin and rutin, such as anti-inflammatory and antioxidant activities, are largely based on the results of in vitro studies [10,11,12,13,14], in which the concentrations of quercetin chosen for unraveling its intracellular mechanisms might not be physiologically relevant. Therefore, whether these mechanisms are applicable to the in vivo biological processes depends on the disposition of quercetin and rutin in vivo, mainly through their bioavailability and biotransformation. In monogastric animals, quercetin is mostly absorbed in the small intestine, with 17% bioavailability in pigs [15] and 59% in dogs [16], whereas rutin is more absorbable in the large intestine after its glycoside bonds are cleaved by bacterial α-rhamnosidase and β-glucosidase to release quercetin [17,18]. In ruminants, ruminal bacteria-mediated degradation occurs prior to the absorption. The bioavailability of intraruminal quercetin and rutin in nonlactating cows was only 0.1 and 0.5%, respectively [19]. The in vitro incubation of quercetin with the ruminal fluid of nonlactating cows showed that almost 90% of quercetin was degraded during the first 5 h of incubation, producing 3,4-dihydroxyphenylacetic acid (DHPAA) and 4-methylcatechol as the major metabolites from fermentation [20]. However, the formation of these microbial metabolites in vivo and their metabolic fates in dairy cows have not been examined.

To investigate the metabolic fate of rutin in dairy cows, both targeted metabolite and untargeted metabolomic analyses were conducted to profile rutin and quercetin metabolites in plasma, urine, and rumen fluids after intraruminal dosing of rutin, as well as the associated metabolic changes in these biological fluids. The distribution of these metabolites was defined by the quantitative analysis. The acquisition of new knowledge on rutin biotransformation may improve our understanding on the health and performance benefits of feeding flavonol supplements or flavonol-rich ingredients to dairy cows.

## 2. Results

### 2.1. Identification of Plasma Metabolites Affected by Intraruminal Rutin

**Figure 1:**
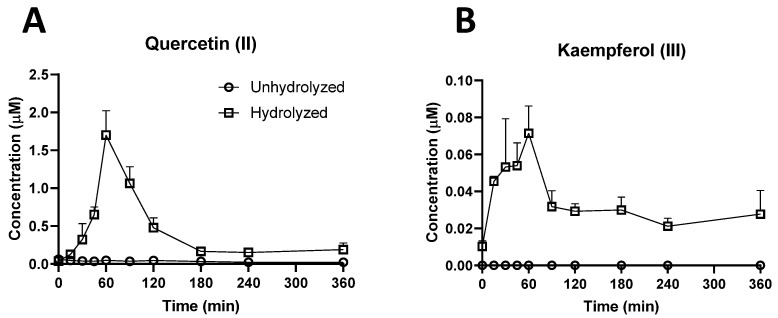
Concentrations of detectable flavonol metabolites of rutin in hydrolyzed (open box) and unhydrolyzed (open circle) plasma samples. Acid hydrolysis was conducted to release flavonols from their respective conjugates in plasma. (**A**) Time course of quercetin (II), and (**B**) time course of kaempferol (III). The Roman numerals (II and III) refer to the ion IDs in Table 1. The concentrations are expressed as means ± SEMs.

A targeted analysis was conducted to determine the presence, as well as the concentrations, of rutin, quercetin, and their known flavonol metabolites, including kaempferol (a dehydroxylated metabolite of quercetin), isorhamnetin, and tamarixetin (two methylated metabolites of quercetin), in both unhydrolyzed and hydrolyzed plasma samples. Rutin (I), isorhamnetin, and tamarixetin were not detected in the samples (data not shown). Quercetin (II) and kaempferol (III) were detected in hydrolyzed samples but not in unhydrolyzed samples (Table 1), indicating extensive conjugation. Moreover, the concentration of kaempferol was more than one order lower than that of quercetin, indicating its status as a minor metabolite (Figure 1A,B). The pharmacokinetics analysis on the time course of quercetin revealed its low plasma concentration (*C_max_* = 1.70 µmol/L), short half-life (*t*_1/2_ = 12 min), high clearance (*Cl* = 0.97 L/min/kg), and large volume of distribution (*V_d_* = 129.63 L/kg) (Table 2).

**Figure 2:**
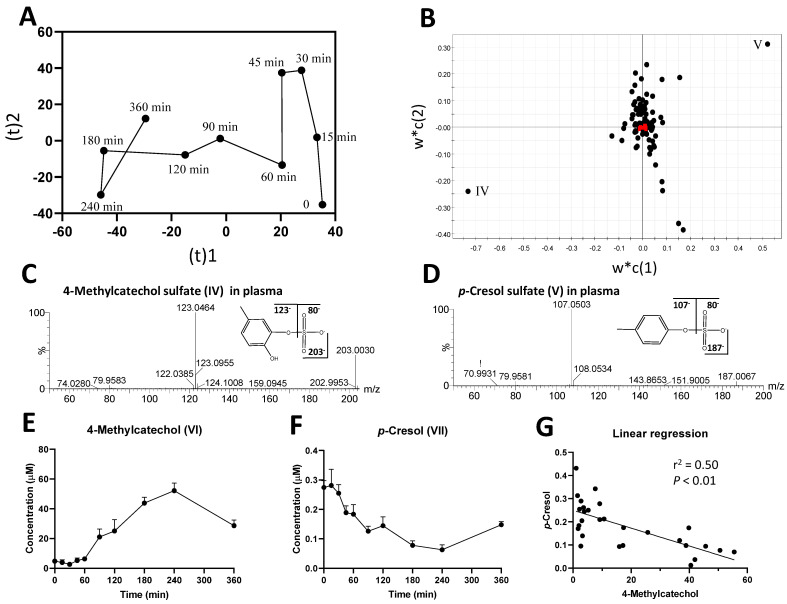
Identification and characterization of the most prominent changes in the plasma metabolome after the intraruminal rutin administration. (**A**) A scores plot of the PLS-DA model on plasma metabolome. The trajectory of treatment-elicited changes is comprised of the means of four samples at each time point in principal components 1 and 2. The distribution of individual samples is presented in Figure S1. (**B**) Loadings plot of the PLS-DA model. The two most prominent metabolites contributing to the separation of samples were labeled (IV–V), and their identities are listed in Table 1. (**C**) MS/MS fragmentogram of 4-methylcatechol sulfate (IV). (**D**) MS/MS fragmentogram of *p*-cresol sulfate (V). (**E**) Time course of 4-methylcatechol (VI), and (**F**) time course of *p*-cresol (VII) after acid hydrolysis procedure. The concentrations are expressed as means ± SEMs. (**G**) Pearson correlation and linear regression analyses of 4-methylcatechol and *p*-cresol in plasma.

To determine whether other quercetin metabolites were also present, an untargeted metabolomics analysis was conducted on the plasma LC-MS data. In the scores plot of a PLS-DA model, the time-dependent separation of plasma samples is observed along with the principal component 1 of the model (Figure 2A and Figure S1 of Supplementary Materials). Two ions (IV–V) contributing to the sample separation through their high values in the principal component 1 were identified in the loadings plot of the model (Figure 2B), and then determined as 4-methylcatechol sulfate (IV) and *p*-cresol sulfate (V) by elemental composition analysis and MS/MS fragmentation (Figure 2C,D, Table 1). After removing the sulfate group from these two metabolites by acid hydrolysis, the concentrations of 4-methylcatechol (VI) and *p*-cresol (VII) in plasma samples were quantified. The results showed that, after the intraruminal dosing of rutin, the concentration of 4-methylcatechol increased gradually and peaked at 240 min, whereas the concentration of *p*-cresol decreased through 240 min (Figure 2E,F). The Pearson correlation analysis showed a significant inverse correlation between these two metabolites (Figure 2G), implying that the formation of 4-methylcatechol sulfate (IV) might negatively affect the production of *p*-cresol sulfate (V).

### 2.2. Identification of Urinary Metabolites Affected by Intraruminal Rutin

**Figure 3:**
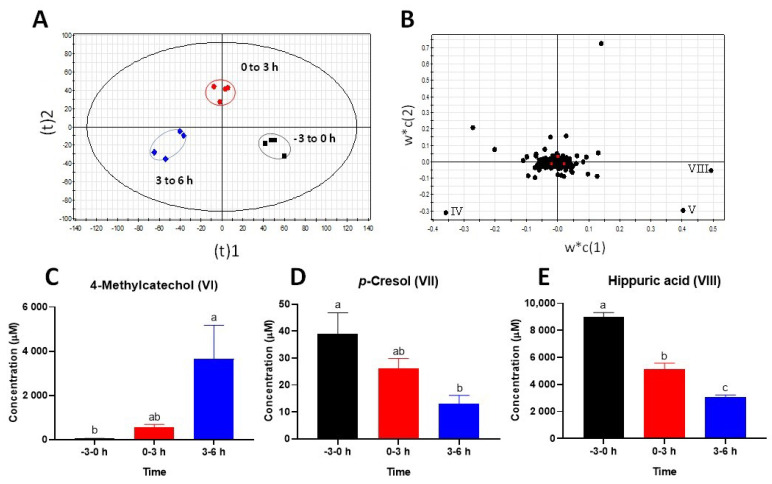
Identification and characterization of the most prominent changes in the urine metabolome after the intraruminal rutin administration. The LC-MS data of pre- and post-treatment of urine samples were processed by PLS-DA modeling. (**A**) A scores plot of the PLS-DA model. Three sample groups (*n* = 4/group) were circled. (**B**) Loadings plot of the PLS-DA model. Three most prominent metabolites contributing to the separation of samples were labeled (IV, V, and VIII), and their identities are listed in Table 1. Concentration of (**C**) 4-methylcatechol (VI), (**D**) *p*-cresol (VII), and (**E**) hippuric acid (VIII) in urine. The concentrations are expressed as means ± SEMs. Different letters indicate significant difference (*p* < 0.05) among timepoints.

The metabolomic analysis of pre-dosing (−3 to 0 h) and post-dosing urine samples (0 to 3 h and 3 to 6 h) showed the time-dependent separation in the scorings plot of a PLS-DA model, mainly along with the principal component 1 (Figure 3A). Three most prominent metabolites (IV–V, VIII) contributing to this separation were identified in the loadings plot of the model through their high values in the principal component 1 (Figure 3B) and then determined as 4-methylcatechol sulfate (IV), *p*-cresol sulfate (V), and hippuric acid (VIII) (Table 1). Consistent with the results of plasma analysis, the analysis of hydrolyzed urine samples showed that the concentration of 4-methylcatechol (VI) increased dramatically while the concentration of *p*-cresol (VII) decreased after the intraruminal rutin dosing (Figure 3C,D). In addition, hippuric acid (VIII) in urine was also decreased by the rutin treatment (Figure 3E).

### 2.3. Investigation of Ruminal Degradation of Rutin and Quercetin

**Figure 4:**
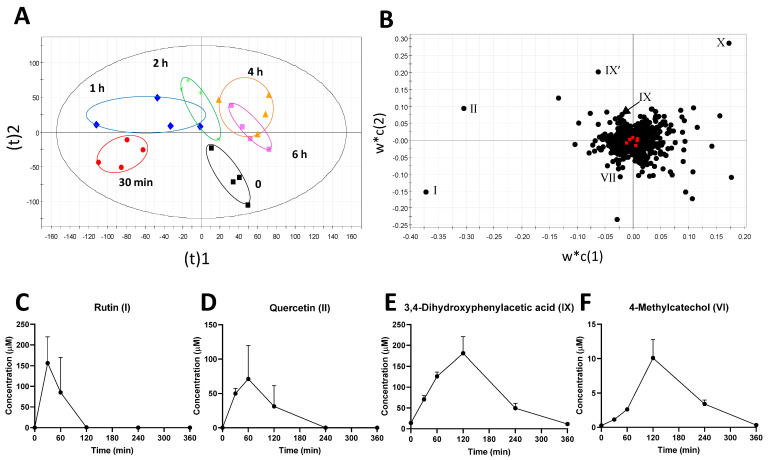
Identification and characterization of the most prominent changes in the rumen metabolome after the intraruminal rutin administration. Data from LC-MS analysis of rumen fluid extracts were processed by PLS-DA modeling. (**A**) Scores plot of the PLS-DA model on rumen metabolome. The samples in the same time point (*n* = 4) were circled. (**B**) Loadings plot of the PLS-DA model. The labeled markers (I, II, VII, IX, IX, and X) are the identified metabolites that contribute to the separation of samples. Their identities are listed in Table 1. Time course of (**C**) rutin (I), (**D**) quercetin (II), (**E**) 3,4-dihydrophenylacetic acid (IX), and (**F**) 4-methylcatechol (VI) in rumen fluid. The concentrations are expressed as means ± SEMs.

The sources and causes of observed metabolic changes in plasma and urine were further examined by the metabolomic analysis of the rumen fluid samples collected at multiple time points after the rutin administration. A distinctive time-dependent separation was observed in the scores plot of a PLS-DA model, in which the trajectory of sample groups showed the reversible metabolic changes in rumen fluid after the rutin administration (Figure 4A). The metabolites contributing to these time-dependent changes were further identified in the loadings plot (Figure 4B), and determined as rutin (I), quercetin (II), DHPAA (IX and IX from its in-source fragment), and 4-hydroxyphenylacetic acid (X). Quantitative analyses of these metabolites in rumen fluid showed that the concentration of rutin (I) decreased rapidly after 30 min and disappeared at 2 h (Figure 4C), while the concentration of quercetin (II) peaked at 1 h and disappeared at 4 h (Figure 4D). Compared to rutin and quercetin, the time course of DHPAA (IX) was further delayed since its concentration peaked at 2 h around 200 µM and returned to its basal level at 6 h (Figure 4E). The targeted analysis of 4-methycatechol (VI) showed a profile similar to DHPAA, but in much lower concentrations (Figure 4F). The half-life (*t*_1/2_) of rutin (I), quercetin (II), DHPAA (IX), and 4-methycatechol (VI) were determined as 10.89 min, 14.62 min, 60.65 min, and 48.22 min, respectively (Table 3). All these profiles indicated a rapid degradation of rutin and quercetin, followed by gradual formation of DHPAA and 4-methylcatechol in the rumen.

### 2.4. Influence of Rutin on Ruminal Tyrosine Metabolism

**Figure 5:**
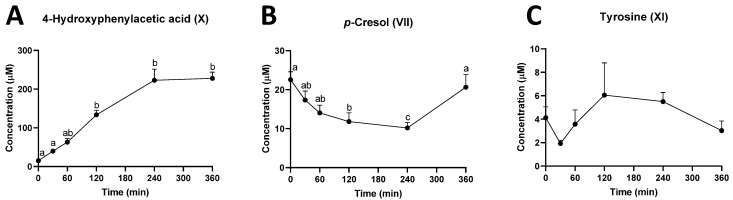
Concentrations of *p*-cresol and its precursors in rumen fluid. Time course of (**A**) 4-hydroxyphenylacetic acid (X), (**B**) *p*-cresol (VII), and (**C**) tyrosine (XI). The concentrations are expressed as means ± SEMs. Different letters (a, b, c) indicate significant difference (*p* < 0.05) among timepoints.

In addition to rutin and quercetin metabolites, 4-hydroxyphenylacetic acid (X), the intermediate metabolite in the microbial conversion of tyrosine to *p*-cresol, was identified as a prominent ruminal metabolite affected by the rutin treatment (Figure 4B). The quantitative analysis showed that the concentration of 4-hydroxyphenylacetic acid continuously increased in the first 4 h of rutin administration and then plateaued afterwards (Figure 5A). In contrast, the concentration of *p*-cresol (VII) gradually decreased in the first 4 h of rutin treatment (Figure 5B). Moreover, the concentration of tyrosine (XI) was not altered by ruminal rutin (Figure 5C). All these observations indicated that the decrease in *p*-cresol might be caused by the inhibition of the conversion from 4-hydroxyphenylacetic acid to *p*-cresol, instead of the deficiency in tyrosine.

### 2.5. Ruminal Short-Chain Fatty Acids (SCFAs)

**Figure 6:**
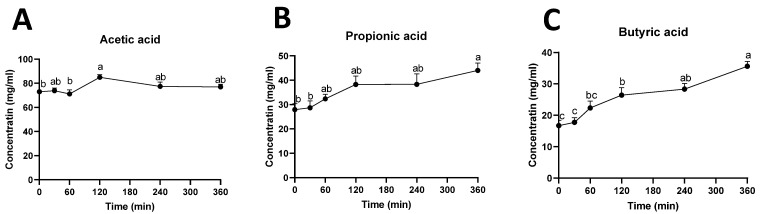
Concentrations of SCFAs in rumen fluid. Time course of (**A**) acetic acid, (**B**) propionic acid, and (**C**) butyric acid. The concentrations and ratio are expressed as means ± SEMs. Different letters (a, b, c) indicate significant difference (*p* < 0.05) among timepoints.

The influences of rutin treatment on rumen fermentation were further examined by measuring the concentrations of SCFAs in the rumen fluid. The results showed that intraruminal rutin had limited effects on acetic acid (Figure 6A), while it consistently increased the concentrations of propionic acid and butyric acid (Figure 6B,C).

## 3. Discussion

**Figure 7:**
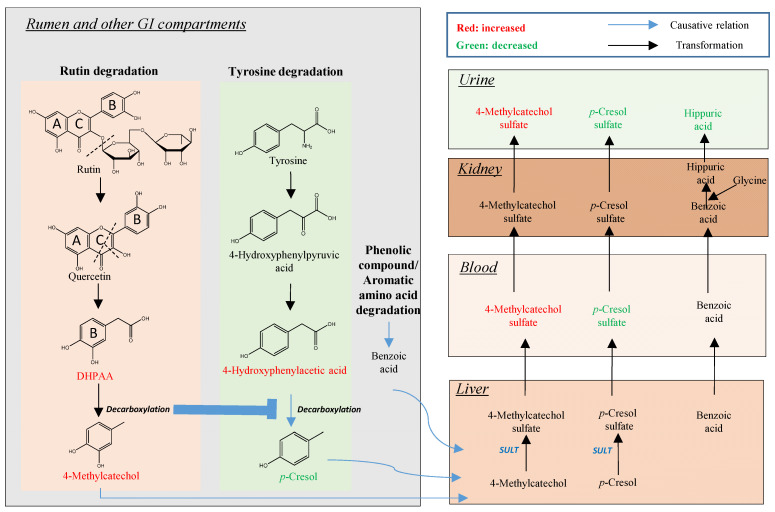
Summary of intraruminal rutin-induced metabolic changes in dairy cow. Microbial metabolites, 4-methylcatechol and *p*-cresol are the bioavailable end products of rutin and tyrosine, respectively. Since these two degradation pathways share the same decarboxylation reaction, the conversion of DHPAA to 4-methylcatechol could inhibit the conversion of 4-hydroxyphenylacetic acid to *p*-cresol. These metabolic events in the rumen further affect the distribution of other rutin and tyrosine metabolites in the plasma and urine of dairy cows.

The identification of 4-methylcatechol, a microbial metabolite, as the dominant bioavailable metabolite of intraruminal rutin, and the inhibitory effect of the rutin → quercetin → DHPAA → 4-methylcatechol degradation route on the microbial production of *p*-cresol, are the two most notable observations from the intraruminal rutin treatment in the current study. The metabolic events associated with these observations occurred either in sequence or in parallel, through both microbial and endogenous metabolism at different physiological sites (Figure 7). The causes and significances of these metabolic events are discussed in the following sections.

### 3.1. 4-Methylcatechol as the Most Bioavailable Metabolite of Rutin and its Significance

Both 4-methylcatechol (3,4-dihydroxytoluene) and its immediate precursor, DHPAA, are known microbial metabolites of rutin [21]. However, our data are the first to demonstrate 4-methylcatechol as the most bioavailable metabolite from microbial metabolism of rutin in dairy cows.

#### 3.1.1. Bioavailability of Rutin, Quercetin, and their Derivatives

Previous bioavailability studies on rutin and quercetin, which were mainly conducted in human and monogastric animals, focused on the post-absorption presence of quercetin and its flavonol metabolites produced via methylation (isorhamnetin and tamarixetin) or dehydroxylation (kaempferol). In dairy cows, the peak plasma concentration of total flavonols, which included quercetin, isorhamnetin, tamarixetin and kaempferol, was around 1 µM from intraruminal administration of 100 mg rutin/kg BW [19]. With the same dose of rutin, the peak plasma concentration of quercetin (1.7 µM) in our study was in the comparable range. In contrast, concentrations of 4-methylcatechol (50 µM) in plasma and urine (3000 µM) were much greater than any other metabolite of rutin. This observation clearly indicates that 4-methylcatechol is the most abundant bioavailable metabolite of intraruminal rutin in dairy cows. In addition, this phenomenon might not occur solely in ruminants since the plasma and urinary concentrations of 4-methylcatechol sulfate and catechol sulfate, another microbial metabolite of flavonols, in human subjects were also 1–3 orders greater than the concentrations of their flavonol precursors [22]. Therefore, more studies are needed to determine the status of 4-methylcatechol and other microbe-derived phenolics as the bioavailable metabolites of flavonoids in ruminants and non-ruminants.

#### 3.1.2. Significance of 4-Methylcatechol as the Dominant Bioavailable Rutin Metabolite

A direct implication of this observation is to provide additional explanations for the documented bioactivities of rutin and quercetin, such as antioxidant and other health- and performance-promoting activities in dairy cows [4,5,6,7]. A previous comparison on the antioxidant activities of quercetin and its metabolites indicated that 4-methylcatechol performed as a robust radical scavenger in the 2,2-diphenyl-1-picrylhydrazyl (DPPH) assay and was equally effective as quercetin for suppressing malondialdehyde production in a cell-based lipid peroxidation assay [23]. Therefore, 4-methylcatechol might alleviate the oxidative stress occurred in the metabolic disorders of periparturient cows, such as hepatosteatosis and ketoacidosis [24,25]. In addition to its antioxidant activity, 4-methylcatechol has been shown to possess strong antiplatelet and hypotensive activities [26,27] and thus could function prophylactically against circulatory morbidities in dairy cows, such as pulmonary artery hypertension-elicited right-heart failure in neonatal calves [28]. It will be interesting to examine whether rutin supplementation might decrease the mortality associated with pulmonary artery hypertension in calves and improve the performance of adult cows.

In the current study, 4-methylcatechol was primarily detected as its sulfate conjugate in plasma and urine. This observation is expected, since sulfation is the most common conjugation reaction for absorbed catechols and phenols (Figure 7). If the non-conjugated form is required for the bioactivities, then hydrolysis of 4-methylcatechol sulfate to release 4-methylcatechol is required prior to or at the site of action. One possible route of deconjugation is through the activity of lysosome sulfatase [29]. Further examination on the status of 4-methylcatechol in bovine tissue may provide more insight on this issue.

### 3.2. Rutin Degradation Pathway and its Interactions with Tyrosine Degradation in the Rumen

The stepwise degradation of rutin → quercetin → DHPAA → 4-methylcatechol in ruminal fermentation has been shown in vitro [20]. The results of our in vivo study provided solid evidence on this pathway through the kinetic profiles of these four compounds (Figure 4 and Table 3). More importantly, this degradation pathway inhibited the degradation of tyrosine to *p*-cresol in rumen (Figure 5). These metabolic reactions and interactions derived from ruminal microbes and their enzymes could have significant impacts on the performance of dairy cows.

#### 3.2.1. Rutin and the Bacteria Responsible for Rutin Degradation

Rapid degradation of rutin to quercetin in the rumen is expected, since many ruminal bacteria, such as *Selenomonas*, *Butyrivibrio*, and *Peptostreptococcus* species, contain glycosidases, a family of enzymes capable of hydrolyzing two glycosidic bonds in rutin [30]. The sugars released by this hydrolysis, including rhamnose, glucose, and rutinose, are available for ruminal fermentation to produce SCFAs, such as *Butyrivibrio*-mediated butyric acid production [31]. The fermentation of rutin-derived sugars, together with the fermentation of recently consumed TMR, likely contributed to the selective increases of ruminal SCFAs in this study. Quercetin can undergo the hydrolytic cleavage of its heterocyclic C ring, a reaction conducted by *Butyrivibrio* and *Clostridium* species [32,33], to form phloroglycinol, an A ring-derived metabolite, and DHPAA, a B ring-derived metabolite (Figure 7). Phloroglucinol can proceed through a series of reduction and oxidation reactions to produce butyric acid and acetic acid, which has been observed in *Eubacterium oxidoreducens* [34], while DHPAA undergoes a decarboxylation reaction to form of 4-methylcatechol, a terminal product from the microbial degradation of rutin [35].

#### 3.2.2. Influence of Rutin Degradation on *p*-Cresol Production and its Potential Mechanism

As an end product of microbial tyrosine degradation, *p*-cresol can be formed directly by cleaving the Cα–Cβ bond of tyrosine, a reaction catalyzed by tyrosine lyase, or by the decarboxylation of 4-hydroxyphenylacetic acid, which is formed by the transamination of tyrosine and oxidation of 4-hydroxyphenylpyruvic acid [36]. After absorption, *p*-cresol is conjugated to its sulfate or glucuronide forms by colonocytes or hepatic cells [37]. The inverse correlation between 4-methylcatechol and *p*-cresol observed in this study (Figure 2G) indicates the potential competition between rutin degradation and *p*-cresol production, especially in the decarboxylation step. This conclusion is supported by the fact that the conversion of DHPAA to 4-methylcatechol and the conversion of 4-hydroxyphenylacetic acid to *p*-cresol occur through the same decarboxylation reaction and therefore, likely share the same decarboxylases. More importantly, the decrease of *p*-cresol in rumen fluid was coincident with the increase of 4-hydroxyphenylacetic acid (Figure 5A), which can be considered as a direct consequence of inhibiting the 4-hydroxyphenylacetic acid → *p*-cresol biotransformation. In fact, 4-hydroxyphenylacetic acid decarboxylase, a glycyl radical enzyme, has been purified and cloned from *Clostridium difficile*, a pathogenic bacteria, and other *Clostridium* species [38]. The substrate affinity analysis showed that DHPAA had higher affinity (*K_m_* = 0.5 mM) than 4-hydroxyphenylacetic acid (*K_m_* = 2.8 mM) for this decarboxylase, and DHPAA inhibited the decarboxylation of 4-hydroxyphenylacetic acid with *K_i_* = 0.4 mM [38]. This decarboxylase activity is likely present in many bacterial species, since *p*-cresol is produced as a degradation product of tyrosine by diverse anaerobic bacteria, such as *Clostridium*, *Faecalibacterium*, *Eubacterium*, *Anaerostipes*, *Ruminococcus*, *Bacteroides*, *Bifidobacterium*, and *Coriobacteriaceae* [36]. In rumen of dairy cows, multiple strains of a *Lactobacillus* sp. have been shown to catalyze the formation of *p*-cresol and 4-methylcatechol [38,39].

As with *p*-cresol, urinary hippuric acid was decreased by rutin administration (Figure 3E). This observation was similar to the results of our recent study, in which urinary hippuric acid, together with indoxyl sulfate and phenylacetylglutamine, were decreased by the supplement of green tea polyphenols in human subjects [40]. Benzoic acid, the precursor of hippuric acid, originated from the microbial metabolism of tyrosine, phenylalanine, phenolics, and quinic acid in plants. Therefore, a similar competitive inhibition mechanism could be responsible for the inhibitory effect of rutin on the biosynthesis of hippuric acid.

#### 3.2.3. Significance of Inhibiting *p*-Cresol Production

Unlike other beneficial metabolites produced by bacterial fermentation, *p*-cresol is known for its negative bioactivities, mainly its inhibitory effects on epithelial cell proliferation, mitochondrial bioenergetic activity, and T helper 1 cell-regulated immune response [41,42]. It has been considered as a uremic toxin due to the correlation between serum *p*-cresol sulfate and endothelial damage in kidney disease [43]. In addition, *p*-cresol is widely considered as a prominent volatile organic compound responsible for the odor of concentrated cattle and dairy operations [44]. Therefore, decreased *p*-cresol in rumen fluid and decreased *p*-cresol sulfate in plasma and urine should be considered as beneficial effects of rutin consumption.

## 4. Materials and Methods

### 4.1. Chemicals and Reagents

The chemicals and reagents used in sample preparation, LC-MS analysis, structural confirmation, and quantification are enlisted in Table S1 of Supplementary Materials.

### 4.2. Animals, Experimental Design, and Sample Collection

Animal care and experimental procedures were approved by the University of Minnesota Institutional Animal Care and Use Committee (protocol 2008-38360A, approved on 16 September 2020). The dairy cows were housed in the University of Minnesota dairy cattle teaching and research farm at the St. Paul campus with free access to feed and water. Four multiparous rumen-cannulated Holstein cows in mid-to-late lactation were fed a standard total mixed ration (TMR) formulated to meet the nutritional needs of Holsteins in late lactation (Table S2). The number of the cows in this study was based on the practices in previous studies, in which ruminal biotransformation and plasma kinetics of quercetin were studied using two to six cows [19,20]. Cows had ad libitum access to feed and water throughout the day, except for the periods of feed change and milking (<30 min, twice per day). Cows had no clinical signs of disease or metabolic disorders and appeared healthy throughout the study. On the day of experiment, each cow received a 100 mg/kg BW intraruminal dose of rutin. Rutin was suspended in 300 mL of deionized water and dosed into the rumen through the cannula for about 1.5 h after fresh TMR was provided. The container of rutin suspension was rinsed twice with 200 mL deionized water, which was also added to the rumen. Rumen fluid samples were collected at 0, 0.5, 1, 2, 4, and 6 h of the rutin administration, and immediately placed on ice. Blood samples were collected via jugular catheter using heparinized tubes at 0, 0.25, 0.75, 1.0, 1.5, 2, 3, 4, and 6 h after the rutin administration, and immediately placed on ice until centrifuged at 2000× *g* for 10 min to separate plasma. Urine samples were collected in 19 L plastic pails and then pooled within three periods, including −3 to 0, 0 to 3, and 3 to 6 h of rutin administration, to form three urine samples per cow. Urine samples were immediately placed on ice after the collection. The aliquots of rumen, plasma, and urine samples were stored at −80 °C prior to analysis.

### 4.3. Metabolites Extraction

Rumen fluid samples were prepared by mixing with 50% aqueous acetonitrile (ACN) in 1:10 (*v*/*v*) ratio; plasma samples were deproteinized by mixing with 50% aqueous ACN in 1:5 (*v*/*v*) ratio; urine samples were prepared by mixing with 50% aqueous ACN in 1:4 (*v*/*v*) ratio. The mixtures were centrifuged at 16,000× *g* for 10 min to precipitate the particles and insolubles. The supernatants were used in sample analysis.

### 4.4. Acid Hydrolysis of Conjugated Metabolites

Glucuronide and sulfate metabolites in plasma or urine samples were hydrolyzed following a modified method [45]. Briefly, 120 µL of plasma or urine sample was mixed with 200 µL of 2 mg/mL *tert*-butyl hydroxyquinone (tBHQ) methanol solution and 80 µL of 10 M HCl, and then incubated in a water bath at 90 °C for 2 h. After cooling, the mixture was further mixed with 400 µL of 2 mg/mL tBHQ methanol solution, and then centrifuged at 16,000× *g* for 10 min. To measure the hydroxyl group contained metabolites, a similar procedure for hydrolysis using methanol without tBHQ was conducted as described above. After cooling, the mixture was neutralized with ammonia hydroxide, and then centrifuged at 16,000× *g* for 10 min. The supernatant was used in the subsequent sample preparation and analysis.

### 4.5. Chemical Derivatization

For detecting the metabolites containing amino group (amino acids) and the ones containing hydroxyl group (*p*-cresol), samples were derivatized with dansyl chloride (DC) prior to the LC-MS analysis [46]. Briefly, 5 µL of sample or standard was mixed with 50 µL of 10 mM sodium carbonate solution, 5 µL of 50 µM deuterated l-tryptophan-(indole-*d_5_*) as an internal standard, and 100 µL of 3 mg/mL DC solution dissolved in acetone. The mixture underwent the incubation at 60 °C for 15 min, cooling on ice, and then centrifugation at 16,000× *g* for 10 min, and the supernatant used for LC-MS analysis. For detecting the metabolites containing carboxyl group, the samples were derivatized with 2-2′-dipyridyl disulfide (DPDS), triphenylphosphine (TPP), and 2-hydrazinoquinoline (HQ) prior to the LC-MS analysis [47]. Briefly, 2 µL of sample or standard was added into 100 µL of freshly prepared ACN solution containing 1 mM DPDS, 1 mM TPP, 1 mM HQ, and 100 µM deuterated *d*_4_-acetic acid as the internal standard. The reaction mixture was incubated at 60 °C for 30 min, chilled on ice, and mixed with 100 µL of H_2_O. This mixture was centrifuged at 16,000× *g* for 10 min and the supernatant was used for LC-MS analysis.

### 4.6. LC-MS Analysis

A 5 μL of aliquot prepared from rumen fluid, plasma, or urine was injected into an Acquity ultraperformance liquid chromatography-quadrupole time-of-flight mass spectrometry (UPLC-QTOFMS) system (Waters, Milford, MA, USA), and then separated in a UPLC column in a 10-min run at a flow rate of 0.5 mL/min. Detailed information on LC-MS acquisition conditions is provided (Table S3). The LC eluant was injected into a Xevo-G2-S QTOF mass spectrometry (Waters, Milford, MA, USA) for accurate mass measurement and ion counting. Capillary voltage and cone voltage for electrospray ionization was maintained at 3 kV and 30 kV for positive-mode detection, or at −3 kV and −35 V for negative-mode detection, respectively. The source temperature and desolvation temperature were set at 120 °C and 350 °C, respectively. Nitrogen was used as both cone gas (50 L/h) and desolvation gas (600 L/h), and argon as collision gas. For accurate mass measurement, the mass spectrometer was calibrated with sodium formate solution (range *m*/*z* 50–1000) and monitored by the intermittent injection of the lock mass leucine enkephalin ((M + H)^+^ = *m*/*z* 556.2771 or (M − H)^−^ = *m*/*z* 554.2615) in real time. Additional structural information was obtained by tandem MS (MS/MS) fragmentation with collision energies ranging from 15 to 45 eV. Mass chromatograms and mass spectral data were acquired and processed by MassLynx^TM^ software V4.2 (Waters, Milford, MA, USA) in centroided format.

### 4.7. Targeted/Quantitative Analysis

The concentrations of rutin, quercetin, methylated quercetin, kaempferol, DHPAA, 4-methylcatechol, *p*-cresol, short-chain fatty acids, and amino acids were determined by calculating the ratio between their individual peak areas and the peak area of internal standard and fitting with a standard curve using QuanLynx^TM^ software (Waters, Milford, MA, USA).

### 4.8. Untargeted Multivariate Data Analysis and Marker Characterization

After data acquisition in the UPLC-QTOFMS system, chromatographic and spectral data of samples were deconvoluted by MarkerLynx^TM^ software. A multivariate data matrix containing information on sample identity, ion identity from retention time (RT) and *m*/*z*, and ion abundance, was generated through centroiding, deisotoping, filtering, peak recognition, and integration. The intensity of each ion was calculated by normalizing the single ion counts (SIC) versus the total ion counts (TIC) in the whole chromatogram. The processed data matrix was exported into SIMCA-P+^TM^ software (Umetrics, Kinnelon, NJ, USA), transformed by *Pareto* scaling, and then analyzed by partial least squares discriminant analysis (PLS-DA) on multiple time points of samples after rutin administration. Major latent variables of the multivariate model were defined in a scores scatter plot. The potential metabolite markers were identified by analyzing ions contributing to the principal components and to the separation of sample groups in the loadings scatter plot. The chemical identities of interested compounds were determined by accurate mass measurement, elemental composition analysis, database search using Human Metabolome Database (https://www.hmdb.ca/, accessed on 21 September 2021), and Metlin (https://metlin.scripps.edu/, accessed on 21 September 2021), MSMS fragmentation, and comparisons with authentic standards if available.

### 4.9. Kinetic Analysis

The kinetic parameters of rumen and plasma samples were determined by the extravascular input non-compartmental analysis (Module 101) of SOLVER in Microsoft Excel 2016 version 16.0.5173.1000 (Redmond, WA, USA) [48].

### 4.10. Statistical Analysis

Experimental values were reported as mean ± standard error of the mean (SEM). The statistical significance among samples at different time points was analyzed by one-way ANOVA followed by Tukey’s post hoc test, Pearson correlation, or linear regression using GraphPad Prism version 8.0.2 (GraphPad, Inc., La Jolla, CA, USA). A value of *p* < 0.05 was considered significant.

## 5. Conclusions

Overall, our current study examined the in vivo ruminal rutin degradation process and identified 4-methylcatechol, an end product of microbial metabolism, as the dominant bioavailable metabolite of rutin and quercetin in dairy cows. The formation of 4-methylcatechol inhibited the microbial degradation of tyrosine to *p*-cresol, potentially through competitive inhibition of decarboxylation reactions. Therefore, 4-methylcatechol was likely responsible for or contributed to the many reported bioactivities of rutin and quercetin, while the decrease in *p*-cresol production could also convey benefits to dairy cows and the environment. These new observations warrant further investigations on the mechanisms and potential benefits of rutin supplementation in feed for cattle.

## Figures and Tables

**Table 1:** Identification of plasma, rumen, and urine metabolites in LC-MS analysis. The metabolites were detected in negative mode ((M − H)^−^) or positive mode after the DC derivatization ((M + DC)^+^).

Ion	Sample	Mode of Ion Detection	*m*/*z* of Detected Ion	Identity	Formula	Δppm	Database ID
I	Plasma Rumen	(M − H)^−^	609.1454	Rutin	C_27_H_30_O_16_	−0.3	HMDB0003249
II	Plasma Rumen	(M − H)^−^	301.0351	Quercetin	C_15_H_10_O_7_	1	HMDB0005794
III	Plasma	(M − H)^−^	285.0361	Kaempferol	C_15_H_10_O_6_	−2	HMDB0005801
IV	Plasma Urine	(M − H)^−^	203.0012	4-Methylcatechol sulfate	C_7_H_8_O_5_S	−0.9	HMDB0240459
V	Plasma Urine	(M − H)^−^	187.0064	*p*-Cresol sulfate	C_7_H_8_O_4_S	−0.5	HMDB0011635
VI	Plasma Urine Rumen	(M − H)^−^	123.0448	4-Methylcatechol	C_7_H_8_O_2_	−3	HMDB0000873
VII	Plasma Urine Rumen	(M + DC)^−^	342.1166	*p*-Cresol	C_7_H_8_O	0.6	HMDB0001858
VIII	Urine	(M − H)^−^	178.0506	Hippuric acid	C_9_H_9_NO_3_	1	HMDB0000714
IX	Rumen	(M − H)^−^	167.0343	DHPAA	C_8_H_8_O_4_	−0.6	HMDB0001336
IX’	Rumen	(M − H)^−^	123.0446	DHPAA (fragment)	C_7_H_8_O_2_	0	
X	Rumen	(M + DC)^+^	386.1061	4-Hydroxyphenylacetic acid	C_8_H_8_O_3_	−0.3	HMDB0060390
XI	Rumen	(M + DC)^+^	648.1838	Tyrosine	C_9_H_11_NO_3_	0.8	HMDB0000158

**Table 2:** Kinetic parameters of quercetin in plasma. The values were calculated based on the concentrations of quercetin in hydrolyzed plasma samples.

Parameter	Quercetin
*AUC*_0–*t*_ (µmol/L × min)	143.30
*V_d_* (L/kg)	129.63
*t*_1/2_ (min)	12.24
*C_max_* (µmol/L)	1.70
*t_max_* (min)	60
*Cl* (L/min/kg)	0.97

**Table 3:** Kinetic parameters of rutin, quercetin, and their microbial metabolites in rumen fluid.

	Rutin	Quercetin	3,4-dihydroxyphenylacetic Acid	4-Methylcatechol
*t*_1/2_ (min)	10.89	14.62	60.65	48.22
*C_max_* (µmol/mL)	156.42	71.16	181.21	10.09
*t_max_* (min)	30	60	120	120

## Data Availability

The processed data is contained within the article and supplementary material. The raw data of chromatographic and spectrometric analyses can be requested from the corresponding authors.

## Supplementary Materials

The following are available online at https://www.mdpi.com/article/10.3390/metabo12010016/s1, Figure S1: Scores plot of plasma samples after intraruminal rutin administration; Figure S2: Identification of ruminal degradation of rutin in rumen fluid, Table S1: Source of chemicals and reagents used in chemical analysis, LC-MS analysis, structural confirmation, and quantification, Table S2: Ingredient and nutrient content of the total mixed ration (TMR), Table S3: LC-MS data acquisition condition in a 10-min run.
