# Supplementary material for: Forming 4-Methylcatechol as the Dominant Bioavailable Metabolite of Intraruminal Rutin Inhibits p-Cresol Production in Dairy Cows
Source: Metabolites. 2021 Dec 24;12(1):16. doi: 10.3390/metabo12010016 (PMC8780962; doi:10.3390/metabo12010016)

Forming 4-methylcatechol as the dominant bioavailable metabolite of intraruminal rutin inhibits *p*-cresol production in dairy cows

Yue Guo[1], Wanda J. Weber[2], Dan Yao[1], Luciano Caixeta[3], Noah P. Zimmerman[4], Jesse Thompson[4], Elliot Block[4], Thomas G. Rehberger[4], Brian A. Crooker[2], Chi Chen[1,2*]

[1] Department of Food Science and Nutrition, University of Minnesota, 1334 Eckles Ave., St. Paul, MN 55108, USA; guoxx390@umn.edu (Y.G.); dyao@umn.com (D.Y.); chichen@umn.edu (C.C.)
[2] Department of Animal Science, University of Minnesota, 1364 Eckles Ave., St. Paul, MN 55108; weber023@umn.edu (W.W.); crook001@umn.edu (B.C.)
[3] Department of Veterinary Population Medicine, University of Minnesota, 1365 Gortner Ave., St.Paul, MN 55108; lcaixeta@umn.edu (L.C.)
[4] Church & Dwight Animal and Food Production, Waukesha, WI; Noah.Zimmerman@churchdwight.com (N.Z.); Jesse.Thompson@churchdwight.com (J.T.); Elliot.Block@churchdwight.com (E.B.); Tom.Rehberger@churchdwight.com (T.R.)
*Correspondence: chichen@umn.edu. Tel.: +1-612-624-7704; Fax: +1-612-625-5272.

SUPPLEMENTARY DATA

Table S1. Source of chemicals and reagents used in chemical analysis, LC-MS analysis, structural confirmation, and quantification.

**Chemicals and reagents**	**Vendor**
Acetonitrile (LC-MS grade), Formic acid (LC-MS grade), Hydrochloric acid, Water (LC-MS grade)	Fisher Scientific (Houston, TX)
Amino acid mixture (acidic), Amino acid mixture (basic), *tert*-Butyl Hydroxyquinose (tBHQ), Dansyl chloride (DC), Hippuric acid	Sigma-Aldrich (St. Louis, MO)
3,4-Dihydroxyphenylacetic acid (DHPAA), 2-Hydrazinoquinoline (HQ), Rutin, Triphenylphosphine (TPP)	Alfa Aesar (Tewksbury, MA)
2-2′-Dipyridyl disulfide (DPDS)	MP Biomedicals, LLC (Irvine, CA)
Fatty acids standards (C4-C22)	Nu-Chek Prep, Inc. (Elysian, MN)
Methanol (LC-MS grade)	Avantor performance materials (Radnor, PA)
4-Methylcatechol, Quercetin	Acros Organics (Fair Lawn, NJ)
3-O-methyl Quercetin/Isorhamnetin	Frontier Specialty Chemicals (Logan, UT)
4-O-methyl Quercetin/Tamarixetin	Cayman Chemical Company (Ann Arbor, MI)
Kaempferol	APExBIO (Houston, TX)

Table S2: Ingredient and nutrient content of the total mixed ration (TMR).

Component	Contribution
Ingredient, % of DM	
Corn silage	19.70
Corn, extra fine, rolled	10.60
Corn gluten	4.90
Alfalfa hay, chopped	5.00
Protein mix[1]	8.45
QLF commercial dairy mix[2]	2.75
Cottonseed, Fuzzy	3.20
Megalac[3]	0.40
Nutrient content, DM basis	
DM, %	55.8
Crude protein, %	16.4
Acid detergent fiber, %	17.3
Neutral detergent fiber, %	17.5
Total digestible nutrients, %	77.1
Net energy-lactation, Mcal/kg	1.72
Calcium, %	0.95
Phosphorus, %	0.41
Magnesium, %	0.37
Potassium, %	1.44
Sodium, %	0.50
Iron, ppm	276
Zinc, ppm	86.2
Copper, ppm	17.4
Manganese, ppm	66.5
Molybdenum, %	1.8
Sulfur, %	0.31
Chloride ion, %	0.58
Dietary cation anion difference[4], mEq/kg	227

[1] Extra fine rolled corn, 29.72%; soybean meal 47% protein, 17.50%; canola meal, 12.50%; amino plus (AG Processing, Inc., Omaha, NB), 8.75%; blood meal, 6.25%; calcium carbonate, 5.50%; sodium bicarbonate, 5.00%, distillers grain, 5.00%; WR Elite Dairy Micro (Vita Plus Corporation, Madison, WI), 2.50%; potassium carbonate, 2.00%; UltraMet (Vita Plus Corporation, Madison, WI), 2.00%; sodium chloride, 2.00%; urea 46% N, 1.25%; Rumensin 90 (Elanco Animal Health, Greenfield, IN), 0.03%.

[2] Molasses-based liquid supplement of soluble sugars. Quality Liquid Feeds, Dodgeville, WI.

[3] Arm & Hammer Animal Nutrition, Ewing, NJ.

[4] DCAD = (Na+K) - (Cl+S) according to Goff (2018).

Table S3: LC-MS data acquisition condition in a 10-minute run.

Target compounds	Column type	Mobile phase	MS detection mode
Amino acids (dansylated)	BEH C18	A: 0.1% formic acid in H_2O<br>B: 0.1% formic acid in ACN	Positive
Fatty acids (HQ derivatization)	BEH C18	A: 2 mM NH_4OAc in water with 0.05% CH_3COOH<br>B: 2 mM NH_4OAc in 95% ACN and 5% H_2O with 0.05% CH_3COOH	Positive
Hydrophilic metabolites	BEH Amide	A: 0.1% formic acid in H_2O<br>B: 0.1% formic acid in ACN	Positive and negative
Rutin and quercetin metabolites	BEH C18	A: 0.1% formic acid in H_2O<br>B: Methanol	Negative

Figure S1. Scores plot of plasma samples after intraruminal rutin administration.

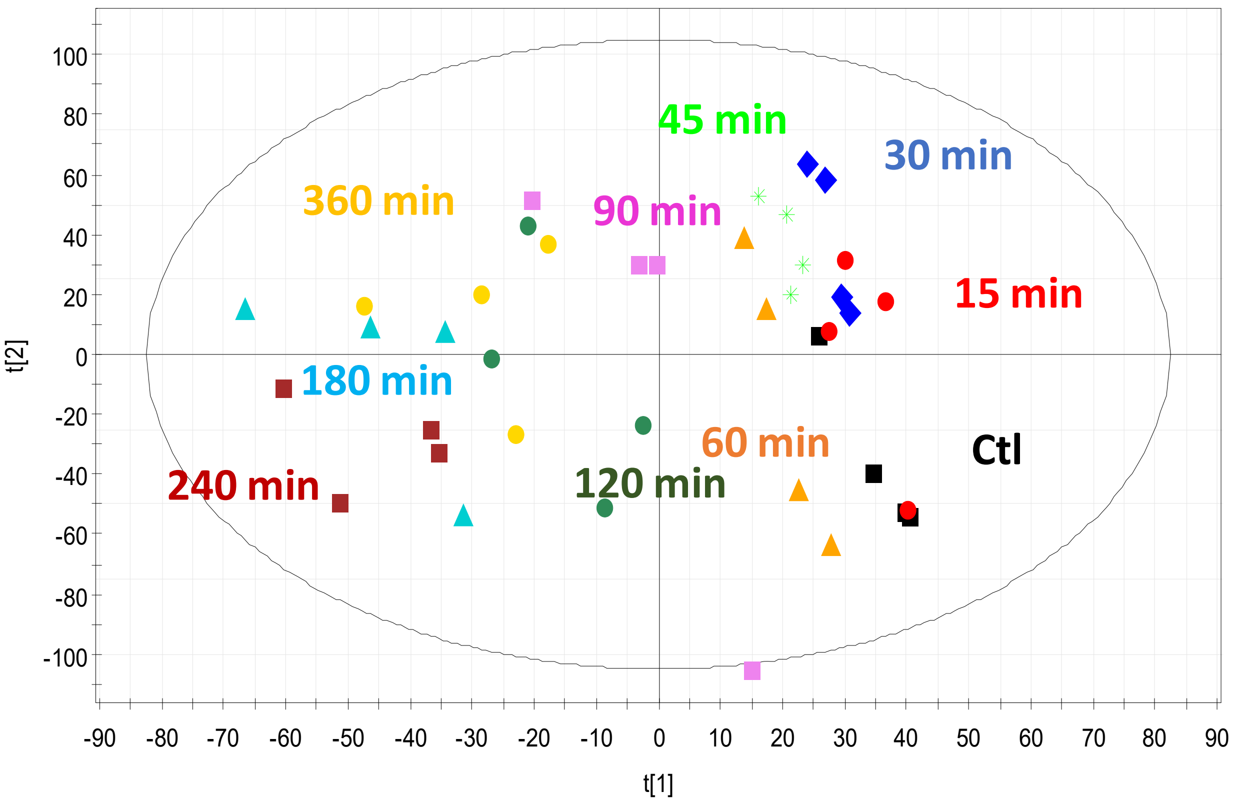

Figure S2. Identification of ruminal degradation of rutin in rumen fluid. **(A)** Extracted chromatography of rutin, quercetin, and DHPAA at 30 min, 1 h and 2 h after intraruminal rutin administration. (B) MS/MS fragmentation of DHPAA standard, and (**C**) 4-methylcatechol standard.

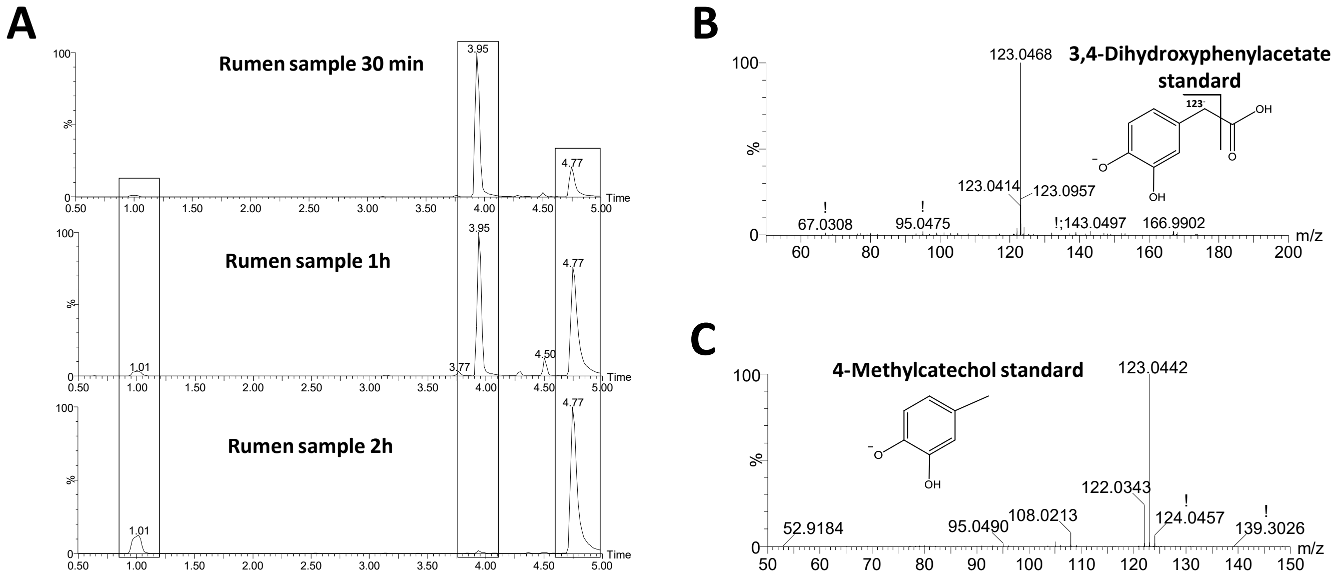

Supplement: Supplementary file 1 [file metabolites-12-00016-s001.zip › metabolites-1524107-supplementary.pdf]
